# P-2265. Treatment Patterns of *Extended-Spectrum Beta-Lactamases (ESBL)-*producing Enterobacterales (ESBLs) In Individuals With Immunocompromised Conditions: Real-World Experiences From An USA Research Network Study

**DOI:** 10.1093/ofid/ofae631.2418

**Published:** 2025-01-29

**Authors:** German Contreras, Masayuki Nigo, Cesar A Arias, Georgy Golovko

**Affiliations:** University of Texas Medical Branch, Houston, Texas; Houston Methodist Hospital, Houston, Texas; Houston Methodist and Weill Cornell Medical College, Houston, TX; University of Texas Medical Branch, Houston, Texas

## Abstract

**Background:**

We sought to characterize the antibiotic preferences, switches, and pathways of treatment of ESBL-producing *Enterobacterales* bacteremia among immunocompromised individuals by using real-world data.

**Figure:**
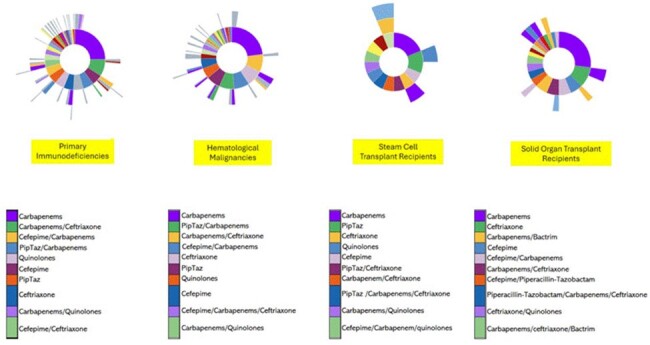
Sunburst diagram of treatment choices for ESBL enterobacterales bacteremia in individuals with immunocompromised conditions

The sunburst illustrates how patients switched between treatments across the lines of antibiotic treatment. The inner ring displays the first line of therapy following the index event. The next ring displays the switch to in the second line of treatment, and so on. The size of each ring corresponds to the proportion patients who followed that pathway. The gray ring color is unknown or other non-listed antibiotic treatment; Carbapenems: includes meropenem, imipenem or ertapenem; Quinolones= includes ciprofloxacin or levofloxacin; PipTaz=piperacillin-tazobactam. The / symbol represents when 2 or more antibiotics were present on the patient’s record for 3 consecutive days.

**Methods:**

We captured treatment patterns within the TrinetX platform for all individuals ≥18 years from January 2020 to December 2023 with the 1^st^ episode of ESBL bacteremia. Four different populations were selected by using ICD-10 codes: 1) Solid Organ Transplant Recipients (SOTr), 2) Steam Cell Transplant Recipients (SCTr), 3) Hematological Malignancies (HM) without transplantation and 4) Primary immunodeficiencies (PI) without transplantation. Treatment patterns were assessed within 7 days following ESBL *enterobacterales* bacteremia diagnosis (index event). The index event was defined based on LOINC codes. A line of treatment was defined as receipt of the same antibiotic within 3 days of index event, and it was considered completed once absent from the patient’s record for 3 consecutive days. The analysis was performed by using the TriNetX package.

**Results:**

We identified 809 individuals with the first episode of ESBL *enterobacterales* bacteremia [PI 390 (48.2%), HM 340 (42%), SOTr 59 (7.3%) and SCTr 20 (2.5%)]

The population was mainly white non-Hispanic and female with average age at index event for PI (59.4 years), HM (68.4) SOTr (59.2) and SCTr (62.9).

The most common initial antibiotic treatment in patients with ESBL bacteremia was carbapenems among all 4 groups.

The average time to carbapenems initiation after index event between groups was [PI (19h±36), HM (19h±40.8) SOTr (36h±58) and SCTr (8h±14).

The average duration of carbapenem therapy was slightly longer in the SCTr (6.6±2.3 days) compared to the other groups [PI (4.3days±2.7), HM (4.2days±2.4) SOTr (4.2days±3.1)]

Other initial antibiotic treatment strategies differed between all groups (Figure). Treatment switches were more frequent in the PI and HM group compared to transplant recipient populations. (Figure)

**Conclusion:**

Our results showed differences in antibiotic therapy practices for ESBL bacteremia across diverse immunosuppressed populations. There is room for improving stewardship interventions in this unique patient population.

**Disclosures:**

All Authors: No reported disclosures

